# From the respective expert viewpoints of the ANM specialty editors

**DOI:** 10.1007/s12149-019-01421-1

**Published:** 2019-11-19

**Authors:** Masayuki Inubushi, Miho Shidahara, Yasuyuki Takahashi, Mikako Ogawa, Yasushi Kiyono

**Affiliations:** 1 Division of Nuclear Medicine, Department of Radiology, Kawasaki Medical School, 577 Matsushima, Kurashiki, 701-0192 Okayama Japan; 2 Department of Quantum Science and Energy Engineering, Graduate School of Engineering, Tohoku University, Aobayama 6-6-01-2, Sendai, 980-8579 Miyagi Japan; 3 Department of Radiation Science, Graduate School of Health Sciences, Hirosaki University, 66-1 Hon-cho, Hirosaki, 036-8564 Aomori Japan; 4 Laboratory for Bioanalysis and Molecular Imaging, Graduate School of Pharmaceutical Sciences, Hokkaido University, N12 W6, Kita-ku, Sapporo, 060-0812 Hokkaido Japan; 5 Biomedical Imaging Research Center, University of Fukui, 23-3 Matsuoka-Shimoaizuki, Eiheiji-cho, Fukui, 910-1193 Japan

**Keywords:** Dosimetry, Harmonic PET, Multimodal imaging, ^18^F-labeled boron-derived methionine analogue (^18^F-B-MET), *meta*-[^211^At]Astato-benzyguanidine (^211^At-MABG)

## Abstract

Although it may not be well known, the Annals of Nuclear Medicine (ANM) Editorial Committee includes one specialty editor of nuclear medicine physics, one of nuclear medicine technology, one of molecular imaging, and two of radiopharmacology. In addition, a statistics editor and a language editor are also on the committee. Manuscripts submitted to ANM can be peer-reviewed by such specialty editors similar to those submitted to highly ranked journals, which is a great pride and joy to us. To offer our readers a condensed global view on the high-quality research work in the field of nuclear medicine, we have published a mini-review article every year under the joint authorship of the ANM associate editors since 2016. This is our fourth serial review article written by the ANM specialty editors from their respective expert viewpoints.

## Introduction

To offer our readers a condensed global view of the high-quality research work conducted in the field of nuclear medicine, we have published a mini-review article every year under the joint authorship of the 5–6 associate editors of the Annals of Nuclear Medicine (ANM) since 2016 [1–3]. This is our fourth serial article written by the final group of 5 authors from about 20 associate editors. Differing from the previous general editor coauthors, the current coauthors are all specialty editors. Although perhaps not well known, the ANM Editorial Committee includes one specialty editor of nuclear medicine physics, one of nuclear medicine technology, one of molecular imaging, and two of radiopharmacology. In addition, a statistics editor and a language editor are also on the committee. Manuscripts submitted to ANM can be peer-reviewed by such specialty editors similar to those submitted to highly ranked journals, which is a great pride and joy to us. In this mini-review article, each ANM specialty editor selects a couple of original articles of their interest from publications in Europe last year, and provide intelligible comments on them from their respective expert viewpoints.

## Nuclear medicine physics–dosimetry in radionuclide therapy

Internal dosimetry of radionuclide therapy is still challenging in clinical practice but essential for individualized treatment planning toward maximum tumor response but minimum normal organ toxicity.

Cremonesi et al. performed an elaborate literature survey of peptide receptor radionuclide therapy (PRRT) with both ^90^Y and ^177^Lu, and reviewed not only the key relationships among absorbed dose, toxicity and tumor response but also the radiobiological models as usually applied for external beam radiation therapy (EBRT) [4]. For kidney toxicity, besides kidney absorbed dose, there are other factors to be considered including the number of cycles, administered activity for each cycle, total administered activity, and amino acid protection. Furthermore, the possibility of renal functional impairment requires a minimum follow-up of 6 months, and more likely 1 year. In EBRT, fractionated irradiation is a common protocol to maximize the tumor response and minimize healthy organ toxicity. As with EBRT, by selecting the optimal number of cycles and administered doses in total or in each cycle, the incidence of kidney toxicity or severity of kidney impairment can be reduced. To accomplish this, the kidney absorbed dose together with dose-limit (e.g., 23 Gy in kidneys, ^177^Lu-DOTATATE, 7.4 GBq [5]) have important roles. Of course, the biological effect factor (BEF) based on the radiobiological model of linear quadrant rather than absorbed dose is sensitive in predicting kidney toxicity. However, the kidney absorbed dose in each cycle will be surely an important factor in routine clinical practice, as mentioned by the authors.

To estimate individual dosimetry, one difficulty is how to obtain an individual time-course of administered biodistributions of administered radioligands in each therapeutic cycle, because therapeutic radionuclides (e.g., alpha emitter) are not always suitable for PET or SPECT imaging. Use of a surrogate nuclide assuming the same biodistribution/biological decay but only different physical decay is one possible solution. Kratochwil et al. performed serial whole-body PET imaging with ^68^ Ga-PSMA-617 as the surrogate radioligand and extrapolated to ^213^Bi-PSMA-617, and then finally estimated the equivalent dose by MIRD and a two spherical model for tumor lesions and salivary glands while considering daughter nuclides (^209^Tl, ^213^Po and ^209^Pb) [6]. Despite their practical approaches, they noted the methodological uncertainty of approx. 20% for the average values of their patients, without referring to individual organ masses. For MIRD, different body weight (and organ masses) as compared with the male adult phantom (74 kg) leads to over- or under-estimation of the kidney-absorbed dose, which also easily applies to many Japanese patients. So far, internal dosimetry of diagnostic-purpose radiopharmaceuticals has been performed for group studies; however, in the case of radionuclide therapy, it would be preferable to obtain dose-estimates individually whenever possible with high accuracy. For more accurate individual internal dosimetry of radionuclide therapy, easy to be introduced in clinical practice but quantitative whole-body imaging techniques (possibly with shorter scan time) would be necessary and the use of individual organ masses/S-values for MIRD may be preferred.

## Nuclear medicine technology–harmonic PET

In PET/CT studies of ^18^F-FDG, scientific societies such as the EANM and SNMMI are closely collaborating to promote standardization of practices, to reduce the variability of quantification in multi-center clinical trials. However, image reconstruction techniques such as time-of-flight (TOF), point spread function (PSF), normalization, randoms, scatter and attenuation corrections in PET systems, and the characteristics of PET scanners such as the type of scintillator, show considerable variation. As a result of these efforts, the use of harmonization is spreading [7]. Harmonization stabilizes SUVs and improves lesion detection while maintaining the performance of many PET systems. Harmonic reconstruction mode employs mean contrast recovery (MCR) and contrast recovery variability. In addition, Gibbs overshoots, partial volume effects, and the reproducibility of signals below 17 mm in diameter are issues that must be addressed in PET images.

This report clearly describes how to improve the quantification that can be achieved with current PET systems [7]. In addition, in examining 18 scanners from three major vendors, most of today's main devices are covered. Phantom study complied with the EANM/EARL guidelines for Image Quality QC. Emission scan times were adjusted by list mode from 5 min per bed position, and 15 reconstruction conditions were consolidated into approximately five. Two or three iterations with 21 or 24 subsets were used for image reconstruction, based on maximum-likelihood and expectation–maximization. The following four points were of particular interest:

1. Before and after the conditions suggested for harmonization, the curvature of SUVmax improved from 0.076–0.290 to 0.212–0.267, and absolute errors of SUVmax improved from 0.157–0.566 to 0.170–0.232.
2. In visual analysis for harmonization, quantitative cutoff criteria were determined based on the bandwidth and characteristics of harmonizing reconstruction modes.
3. SUVpeaks for spheres less than 17 mm in diameter have a low recovery rate, and MCR is significantly (20–40%) lower than SUVmax. By reducing the VOI size, SUVpeak may be become an effective alternative to SUVmax.
4. Using the workstation's post-smoothing function, higher recoveries could reduce noise and Gibbs artefacts to acceptable levels for harmonization.

Previous harmonization was not used to the complexity of including TOF and PSF. In the future, harmonization is also expected to PET systems featuring the current silicon photo multiplier (SiPM) and depth of interaction (DOI) detector.

## Molecular imaging–multimodal imaging

The combination of nuclear and optical imaging should be efficient; however, most studies are limited to animal experiments or small-scale clinical trials. KleinJan GH, et al. have tested indocyanine green (ICG)-^99m^Tc dual-labeled nanocolloid for sentinel lymph node (SN) detection in relatively large clinical studies [8]. ICG is a near-infrared fluorophore that is used for lymphatic imaging in the clinic. In this study, they used not free ICG but ICG-colloid that is also labeled with ^99m^Tc to avoid leakage of the tracer into the surgical field. They compared intraoperative radioguidance, fluorescence guidance with hybrid tracer and optical guidance with blue dye. The find rates of SNs with blue dye were significantly poorer than those of the hybrid tracer. The advantages of fluorescence imaging are ease of use and high resolution during surgery. Fluorescence guidance was superior to radioguidance in anatomically complex areas and for identification of lymph nodes next to the injection site. However, since the permeability of the fluorescence is not sufficient, it was difficult to detect the signal from deep tissue. In this article, it is revealed that SNs located at a depth of 0.5–1 cm could not be detected by fluorescence guidance. Fluorescence guidance is effective, but radioguidance remains essential for in vivo SN localization. This article showed the effectiveness of multimodal imaging in the clinic to take advantage of each imaging modality and compensate for their respective shortcomings.

## Radiopharmacology—novel radiopharmaceuticals for diagnosis and therapy

^11^C-Methionie is a useful PET tracer for detecting and grading gliomas. However, due to its short half-life (20.38 min), extensive application of ^11^C-methionine is limited. Yang et al. have developed ^18^F-labeled boron-derived methionine analogue, ^18^F-B-MET [9]. This tracer successfully distributed in mice glioma, and orthotropic tumors were clearly visualized with low background signal in normal brain. The notable points of this article are not only the efficacy of this tracer as a substitute for ^11^C-methionine, but also the simple radiolabeling method. In this article, ^18^F labeling was performed by ^18^F^-19^F isotope exchange reaction, using ^19^F-B-MET as a precursor. The reaction was performed by just mixing the chemicals including ^18^F-fluoride in an aqueous solution, with a high radiochemical yield and short synthesis time accomplished. The biggest concern was low specific activity. Separation from the precursor is impossible since the precursor and product are chemically identical. But, in this article, specific activity was more than 37 GBq/μmol, which is acceptable for most imaging probes. The reaction was performed with only 10 nmol precursor due to the high reactivity of this method, and thus relatively high specific activity was accomplished. As the authors mention in this article, the isotope exchange ^18^F labeling should not only simplify the methodology, but also provides high yields to lower the bar for various clinical applications.

The development of radiopharmaceuticals for internal radiation therapy (or targeted radionuclide therapy: TNT) has been increasing. From the point of view of therapeutic radionuclides, α-emitters are attractive radionuclides exerting strong therapeutic effects with minimal side effects. ^211^At is one such suitable α-emitter because ^211^At is a halogen and has similar characteristics to those of ^131^I. Oshima, et al. investigated the therapeutic effect of *meta*-[^211^At]astato-benzyguanidine (^211^At-MABG) which was an α-emitting type of *meta*-iodo-benzyguanidine (MIBG) in a pheochromocytoma (PC-12 cells) model both in vitro and in vivo [10]. In this study, they demonstrated that ^211^At-MABG reduced the PC-12 cell survival ratio in a dose-dependent manner, with this cell death induced by DNA double-strand breaks in an in vitro study. In an in vivo study, treatment with ^211^At-MABG reduced the tumor volumes in PC12 tumor-bearing mice in a dose-dependent manner. In addition, reductions in body weight and in the number of myeloid cells in the bone marrow were not severe in mice treated with ^211^At-MABG. This article showed the effectiveness of ^211^At-MABG treatment of malignant pheochromocytoma.

From the point of view of therapeutic targets, angiogenesis is an attractive target because when increased it is a marker of aggressiveness in many cancers. CD105 is an endothelial cell marker that has been suggested to be a suitable biomarker for angiogenesis, and it has a very limited biodistribution in normal tissue. Ehlerding, et al. developed ^177^Lu-DTPA-TRC105 as CD105 targeted radiopharmaceuticals as a widely applicable target for targeted radioimmunotherapy [11]. Using breast cancer model mice, they demonstrated that ^177^Lu-DTPA-TRC105 was steadily accumulated in 4T1 tumors between 1 and 7 days after injection. Furthermore, significant inhibition of tumor growth was observed in a group treated with 11 MBq of ^177^Lu-DTPA-TRC105, with a corresponding significant increase in survival. The body weights of the mice did not significantly decrease, and the blood counts indicated minimal differences in toxicity markers. From these results, ^177^Lu-DTPA-TRC105 would appear to be a promising radiopharmaceutical for targeted angiogenesis.

## Conclusion

The ANM specialty editors of nuclear medicine physics, nuclear medicine technology, molecular imaging, and radiopharmacology provided intelligible comments on research in their respective specialties that has attracted their interest. This mini-review article highlighted mainly basic research articles that have been published in the European Journal of Nuclear Medicine and Molecular Imaging (EJNMMI) in 2018. This world-leading journal also published many outstanding clinical research articles; we suppose that targeted radiotherapy using prostate-specific membrane antigen (PSMA) radioligands in prostate cancer patients [12–17] must be a particular focus of attention for most ANM readers in Japan, who are waiting impatiently for its approval by the Japanese government. This topic will be fully discussed in our next serial review article in the near future.

## References

[1] Inubushi M, Higashi T, Kuji I, Sakamoto S, Tashiro M, Momose M (2016). Introduction of nuclear medicine research in Japan. Eur J Nucl Med Mol Imaging..

[2] Inubushi M, Kaneta T, Ishimori T, Imabayashi E, Okizaki A, Oku N (2017). Topics of nuclear medicine research in Europe. Ann Nucl Med..

[3] Inubushi M, Tatsumi M, Yamamoto Y, Kato K, Tsujikawa T, Nishii R (2018). European research trends in nuclear medicine. Ann Nucl Med..

[4] Cremonesi M, Ferrari ME, Bodei L, Chiesa C, Sarnelli A, Garibaldi C (2018). Correlation of dose with toxicity and tumour response to ^90^Y- and ^177^Lu-PRRT provides the basis for optimization through individualized treatment planning. Eur J Nucl Med Mol Imaging..

[5] Sandstrom M, Garske-Roman U, Granberg D, Johansson S, Widstrom C, Eriksson B (2013). Individualized dosimetry of kidney and bone marrow in patients undergoing ^177^Lu-DOTA-octreotate treatment. J Nucl Med..

[6] Kratochwil C, Schmidt K, Afshar-Oromieh A, Bruchertseifer F, Rathke H, Morgenstern A (2018). Targeted alpha therapy of mCRPC: dosimetry estimate of ^213^Bismuth-PSMA-617. Eur J Nucl Med Mol Imaging..

[7] Kaalep A, Sera T, Rijnsdorp S, Yaqub M, Talsma A, Lodge MA (2018). Feasibility of state of the art PET/CT systems performance harmonisation. Eur J Nucl Med Mol Imaging..

[8] Klein Jan GH, van Werkhoven E, van den Berg NS, Karakullukcu MB, Zijlmans HJMAA, van der Hage JA, van de Wiel BA, Buckle T, Klop WMC, Horenblas S, Olmos VRA, van der Poel HG, van Leeuwen FWB (2018). The best of both worlds: a hybrid approach for optimal pre- and intraoperative identification of sentinel lymph nodes. Eur J Nucl Med Mol Imaging..

[9] Yang X, Liu Z, Zhang H, Li Z, Munasinghe JP, Niu G, Teng G, Chen X (2018). Preclinical evaluation of an ^18^F-trifluoroborate methionine derivative for glioma imaging. Eur J Nucl Med Mol Imaging..

[10] Ohshima Y, Sudo H, Watanabe S, Nagatsu K, Tsuji AB, Sakashita T (2018). Antitumor effects of radionuclide treatment using α-emitting meta-^211^At-astato-benzylguanidine in a PC12 pheochromocytoma model. Eur J Nucl Med Mol Imaging..

[11] Ehlerding EB, Lacognata S, Jiang D, Ferreira CA, Goel S, Hernandez R (2018). Targeting angiogenesis for radioimmunotherapy with a ^177^Lu-labeled antibody. Eur J Nucl Med Mol Imaging..

[12] Rahbar K, Boegemann M, Yordanova A, Eveslage M, Schäfers M, Essler M (2018). PSMA targeted radioligand therapy in metastatic castration resistant prostate cancer after chemotherapy, abiraterone and/or enzalutamide. A retrospective analysis of overall survival. Eur J Nucl Med Mol Imaging..

[13] Rahbar K, Bögeman M, Yordanova A, Eveslage M, Schäfers M, Essler M (2018). Delayed response after repeated ^177^Lu-PSMA-617 radioligand therapy in patients with metastatic castration resistant prostate cancer. Eur J Nucl Med Mol Imaging..

[14] Rahbar K, Ahmadzadehfar H, Boegemann M (2018). ^177^Lu-PSMA-617 radioligand therapy in mCRPC: ready for phase III trial?. Eur J Nucl Med Mol Imaging.

[15] von Eyben FE, Roviello G, Kiljunen T, Uprimny C, Virgolini I, Kairemo K (2018). Third-line treatment and ^177^Lu-PSMA radioligand therapy of metastatic castration-resistant prostate cancer: a systematic review. Eur J Nucl Med Mol Imaging..

[16] Virgolini I, Decristoforo C, Haug A, Fanti S, Uprimny C (2018). Current status of theranostics in prostate cancer. Eur J Nucl Med Mol Imaging..

[17] Kelly J, Amor-Coarasa A, Ponnala S, Nikolopoulou A, Williams C, Schlyer D (2018). Trifunctional PSMA-targeting constructs for prostate cancer with unprecedented localization to LNCaP tumors. Eur J Nucl Med Mol Imaging..

